# Enhanced Leaf Disease Segmentation Using U‐Net Architecture for Precision Agriculture: A Deep Learning Approach

**DOI:** 10.1002/fsn3.70594

**Published:** 2025-07-14

**Authors:** Gurpreet Singh, Asma A. Al‐Huqail, Ahmad Almogren, Sukhdeep Kaur, Kapil Joshi, Ajay Singh, Salil Bharany, Seada Hussen, Ateeq Ur Rehman

**Affiliations:** ^1^ Chitkara University Institute of Engineering and Technology Chitkara University Punjab India; ^2^ Department of Botany and Microbiology, College of Science King Saud University Riyadh Saudi Arabia; ^3^ Department of Computer Science, College of Computer and Information Sciences King Saud University Riyadh Saudi Arabia; ^4^ Department of CSE UIT, Uttaranchal University Dehradun Uttarakhand India; ^5^ School of Applied and Life Sciences Uttaranchal University Dehradun Uttarakhand India; ^6^ Department of Electrical Power Adama Science and Technology University Adama Ethiopia; ^7^ School of Computing Gachon University Seongnam‐si Republic of Korea

**Keywords:** CNN, deep learning, image processing, leaf disease segmentation, plant pathology, precision agriculture, semantic segmentation, U‐Net architecture

## Abstract

This study presents a deep learning‐based image segmentation approach for leaf disease identification using the U‐Net architecture. Convolutional neural networks (CNNs), particularly U‐Net, are effective for precise segmentation tasks and were trained and validated on a high‐quality “Leaf Disease Segmentation” dataset. Each image contains annotated regions of unhealthy leaf tissue, enabling the model to distinguish between healthy and infected areas. Image preprocessing and augmentation further enhanced model performance and robustness. The U‐Net model, composed of an encoder for context extraction and a decoder for precise segmentation was trained to accurately identify diseased regions at the pixel level. Regularization techniques such as dropout, batch normalization, and ReLU activation were used to prevent overfitting and improve learning. Furthermore, Adam optimizer was employed with a learning rate of 0.001. The model demonstrated strong generalization by accurately segmenting disease regions in unseen validation images. It effectively captured complex patterns in both healthy and diseased leaf sections, outperforming traditional image processing techniques. Trained on 7056 images for 40 epochs, the model achieved 99.70% training accuracy, 0.062 training loss, and 98.99% validation accuracy. These results highlight the model's high accuracy, efficient learning, and robustness, making it suitable for real‐world applications in precision agriculture.

## Graphical abstract

This study proposes a deep learning‐based approach for leaf disease identification using the U‐Net architecture for precise segmentation. By training on a dataset of 7056 annotated leaf images, the model effectively distinguishes between healthy and diseased regions, achieving 99.70% training accuracy and 98.99% validation accuracy in 40 epochs. The results demonstrate the model's robustness and potential for real‐world precision agriculture applications.

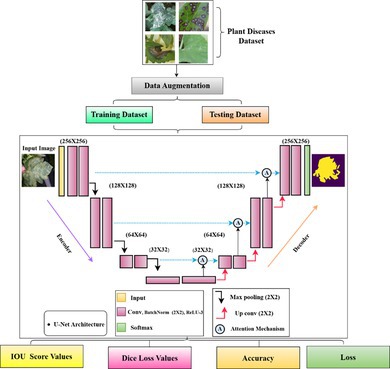

## 1 Introduction

Plant diseases have been on the rise, causing significant challenges for farmers in agriculture. As a result, precision agriculture has become increasingly important as a technological advancement, playing a vital role in improving sustainability and productivity in the field. One of the primary challenges in this area is the early identification and management of plant diseases since this significantly influences crop output and quality. Especially in terms of leaf disease diagnosis, reducing agricultural losses depends much on this. Conventional approaches for disease identification based on human knowledge are time‐consuming, labor‐intensive, and prone to inaccuracy. Consequently, using cutting‐edge image segmentation methods and deep learning (DL) models to automate and raise the accuracy of plant disease diagnosis attracts rapid attention. In this regard, U‐Net, a potent deep‐learning network well‐known for segmentation capabilities, has shown great accuracy and efficiency for leaf disease segmentation.

Several techniques for the classification and identification of leaf diseases have been put forward in previous research. Singh and Misra (2017), the authors showed how image segmentation and soft computing techniques may help identify plant leaf diseases. Their research established a fundamental grasp of the potential use of computer methods in agriculture. By assessing the effect of segmentation on the categorization of wheat stripe rust disease, the research in (Bukhari et al. 2021) expanded on this work. Phan et al. (2022), using DL with simple linear iterative clustering (SLIC), the foliar disease areas on maize leaves were identified. By effectively defining disease‐affected regions in both field and homogenous settings, this approach showed how adaptable segmentation approaches are in various settings. U‐Net is one of the DL architectures that has drawn the most attention due to its exceptional performance in biological image segmentation. It is increasingly used in agricultural contexts to segment and categorize plant leaf diseases. In order to increase segmentation accuracy and classification performance, the study in (Abinaya et al. 2023) suggested integrating a cascade autoencoder with attention residual U‐Net. Similarly, to detect unhealthy areas in crop leaves, the work in (Liu and Zhang 2023) presented a spatial pyramid‐based encoder‐decoder architecture (CCNN). A disease detection network and an area disease segmentation network are the two main components of the network.

The detection module combines a three‐level cascade convolutional neural network (CNN) to extract hierarchical characteristics with a spatial pyramid. The receptive field is enlarged by shared spatial pyramid layers, which enables the model to handle different input sizes. Multi‐scale convolution kernels are used by the segmentation network, which is built on an encoder‐decoder structure, to extract enhanced local features. High segmentation accuracy was shown by experimental findings across various backdrops, maintaining significant morphological features, including color and texture, while attaining a dice similarity coefficient (DSC) of over 95% and other performance metrics surpassing 90%. In (Yuan et al. 2021), the Spatially Pyramid‐Oriented Encoder‐Decoder Cascade Convolutional Neural Network (SPEDCCNN) was unveiled, marking further progress. In order to collect multi‐scale data and improve segmentation accuracy across various plant species and disease kinds, our model used a regional pyramid structure. The technique accurately isolated sick patches of any size or form by integrating spatial pyramids into the encoder‐decoder system. Another contribution was the YR2S model, which was created in Madhurya and Jubilson (2024) and provides a DL framework for the annotation and real‐time detection of plant leaf diseases. Scalability and responsiveness, which are crucial for real‐world implementation in precision agriculture, were highlighted in this model. Even with these noteworthy advancements, there are still difficulties in developing reliable and broadly applicable models for leaf disease segmentation. Conventional image processing methods like GrabCut and Watershed have proven ineffective, particularly when handling occlusions, irregular illumination, and intricate disease patterns. Furthermore, these traditional approaches often fail to retain accuracy when applied to large‐scale datasets comprising various crop species and disease symptoms.

To address these limitations, several studies including (Wang et al. 2023) have explored integrating machine learning techniques and sensor fusion to improve agricultural outcomes. The current work aims to leverage the strengths of DL to achieve accurate and automated leaf disease segmentation. Originally developed for biomedical image segmentation, U‐Net is increasingly recognized for its capacity to generate high‐quality segmentation maps and capture intricate details. Its symmetric encoder‐decoder architecture with skip connections enables the model to simultaneously learn global context and fine‐grained features. It is particularly suitable for handling the complex visual patterns typical of diseased leaf regions.

The key contributions of this study are outlined as follows:

- This study incorporates attention mechanisms and identity mappings into the classical U‐Net, enabling precise segmentation even under challenging visual conditions.
- Additionally, Dice + BCE loss has been added to address class imbalance and improve segmentation accuracy.
- Designed to detect infected regions without requiring class‐specific labels, enabling early‐stage and class‐agnostic disease identification.

## 2 Related Literature

Numerous studies have focused on improving plant leaf disease detection using segmentation‐based techniques combined with various machine learning and DL methods. The relevant literature supporting this study is summarized in Table 1. It highlights the key research works in the field of plant leaf disease detection and segmentation. Singh and Misra (2017) were among the early contributors, implementing image segmentation with genetic algorithms on a small dataset of 106 images. Their approach, which achieved 95.60% accuracy, highlighted the potential of soft computing techniques for early‐stage plant disease detection. Bukhari et al. (Bukhari et al. 2021) studied the effect of segmentation on disease classification accuracy by combining U2‐Net and ResNet18 on a dataset of 5320 wheat stripe rust images, attaining an accuracy of 92.40%. Their work confirmed the importance of accurate segmentation in enhancing classification outcomes. Similarly, Phan et al. (2022) addressed real‐world variability by applying SLIC segmentation and DL to corn leaf datasets captured under field and lab conditions. Their method performed well in such diverse settings, achieving 94.80% accuracy. Abinaya et al. (2023) proposed a hybrid architecture combining an Attention Residual U‐Net with an autoencoder, using a multiclass leaf disease dataset of 9600 images. With an accuracy of 96.30%, their model effectively leveraged attention mechanisms and residual learning to boost segmentation accuracy. Yuan et al. (2021) introduced a spatial pyramid encoder‐decoder cascade CNN model trained on 8250 images, achieving 95.90% accuracy. This architecture enabled multiscale feature extraction for precise segmentation. Madhurya and Jubilson (2024) presented YR2S, a lightweight yet accurate DL framework that achieved 98.10% accuracy on 12,000 plant disease images, making it ideal for resource‐constrained deployment scenarios.

**TABLE 1:** Comparison of state of the art.

Ref	Study focus	Method used	Dataset name	Number of images	Accuracy	Key contribution
Singh and Misra (2017)	Detection of plant leaf diseases using segmentation and soft computing	Image segmentation + genetic algorithm	Self‐collected plant dataset	106	95.60%	Early integration of soft computing for plant disease detection
Bukhari et al. (2021)	Impact of segmentation on wheat stripe rust classification	U2‐Net + ResNet18	Wheat stripe rust dataset	5320	92.40%	Analyzed segmentation impact on classification accuracy
Phan et al. (2022)	Disease region identification on corn leaves in field conditions	SLIC segmentation + deep learning	Corn leaf dataset (field + lab)	10,000	94.80%	The proposed method is effective under real‐world field conditions
Abinaya et al. (2023)	Multi‐class leaf disease segmentation and classification	Attention Residual U‐Net + autoencoder	Custom multi‐class plant dataset	9600	96.30%	Introduced hybrid model with attention and residual features
Yuan et al. (2021)	Leaf segmentation using spatial pyramid encoder‐decoder CNN	Encoder‐decoder cascade CNN	Crop disease leaf dataset	8250	95.90%	Proposed a spatial pyramid encoder‐decoder architecture
Madhurya and Jubilson (2024)	Efficient DL method for plant leaf disease classification	Efficient deep learning with YR2S	Benchmark plant disease dataset	12,000	98.10%	Proposed lightweight and accurate classification framework
Wang et al. (2023)	Pear leaf disease segmentation in natural conditions	MFBP‐UNet	Pear leaf dataset (natural scenes)	2000	98.60%	Proposed MFBP‐UNet for robust segmentation in real farm conditions
El‐Assiouti et al. (2023)	Fast image super‐resolution and segmentation for plant leaf diseases	Lite‐UNet + Lite‐SRGAN	Public leaf image dataset	12,000	98.10%	Achieved efficient segmentation with lightweight models
Bhatti et al. (2024)	Plant disease segmentation in precision agriculture	Fuzzy C‐means + Deep Learning	Precision agriculture dataset	18,000	97.90%	Integrated fuzzy clustering to enhance segmentation precision
Dayang and Kouyim Meli (2021)	Comparison of segmentation algorithms for disease detection	(GLCM) and classification (SVM)	Multiple segmentation algorithm datasets	8000	94.50%	Benchmarked multiple segmentation methods
Mzoughi and Yahiaoui (2023)	Segmentation for plant disease identification	FCN, PSPnet and UNET	Plant leaf dataset	6500	96.20%	Validated segmentation effectiveness with deep learning
Zhang et al. (2019)	Leaf segmentation using superpixel clustering and EM	Superpixel clustering + EM algorithm	Leaf disease dataset	5000	93.80%	Used clustering and EM for improved boundary detection
Elangovan and Nalini (2017)	Plant disease classification using segmentation and SVM	Image segmentation(Otsu method, k‐means clustering) + SVM	Local plant dataset	4500	91.40%	Combined segmentation and classification for plant diseases
Zhang and Zhang (2023)	Modified U‐Net for leaf disease segmentation	Modified U‐Net architecture	PlantVillage subset	7200	97.50%	Enhanced U‐Net with structural modifications for better segmentation

Wang et al. (2023) developed MFBP‐UNet, specifically designed to work in natural agricultural environments. Their model was evaluated on 2000 pear leaf images and yielded the highest accuracy among the reviewed studies at 98.60%. El‐Assiouti et al. (2023) proposed a lightweight solution by integrating Lite‐UNet with Lite‐SRGAN for both image super‐resolution and segmentation, achieving 98.10% accuracy on a public dataset of 12,000 images. Bhatti et al. (2024) used fuzzy C‐means clustering combined with DL on a large‐scale precision agriculture dataset (18,000 images), reporting 97.90% accuracy and highlighting the benefits of dimensionality reduction in enhancing segmentation performance. In terms of benchmarking classical methods, Dayang and Kouyim Meli (2021) conducted a comparative study using GLCM for feature extraction and SVM for classification across 8000 plant disease images, reaching 94.50% accuracy. Mzoughi and Yahiaoui (2023) evaluated the effectiveness of multiple DL architectures, including FCN, PSPNet, and U‐Net, on 6500 images, achieving 96.20% accuracy. *Zhang* et al. (2019) employed superpixel clustering combined with the Expectation–Maximization algorithm to segment diseased areas, achieving 93.80% accuracy on 5000 images. Elangovan and Nalini (2017) used a more traditional pipeline—Otsu thresholding and k‐means clustering followed by SVM—on a local dataset of 4500 images, resulting in 91.40% accuracy. Finally, Zhang and Zhang (2023) proposed structural modifications to the standard U‐Net architecture, applying it to a subset of the PlantVillage dataset with 7200 images. Their improved model achieved 97.50% accuracy and demonstrated better segmentation fidelity, particularly in detecting small or subtle disease regions. Collectively, these studies showcase the evolution from classical machine learning techniques to hybrid DL models, emphasizing the importance of model architecture, dataset scale, and environmental variability in achieving accurate plant disease segmentation.

## 3 Materials and Methods

**FIGURE 1:**
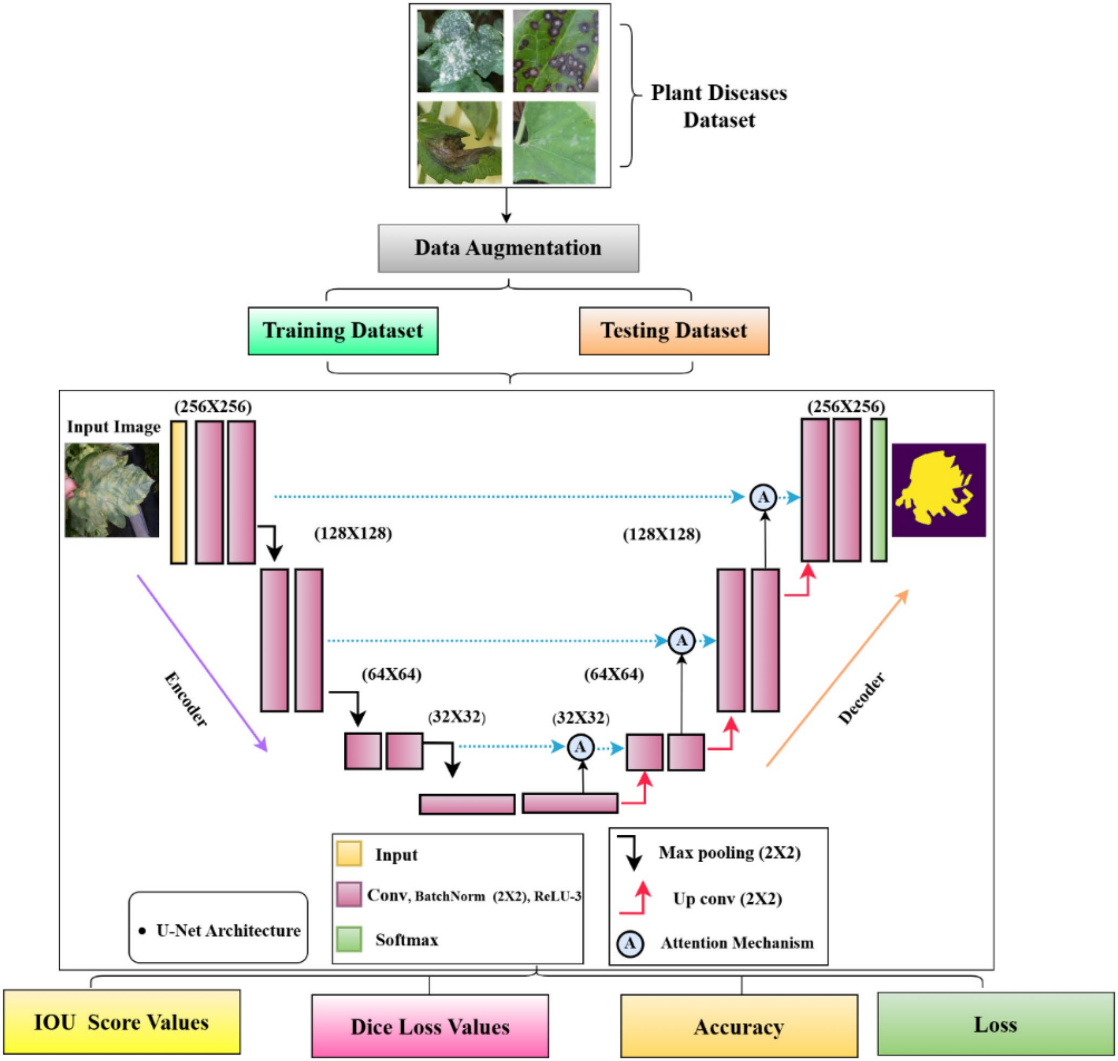
Proposed methodology for plant leaf diseases segmentation.

This section describes the dataset characteristics, preprocessing steps, data augmentation strategies, architectural design of the proposed hybrid U‐Net model, integrated with attention mechanism training procedures, and evaluation metrics. The study utilized a mixed dataset sourced from Kaggle, comprising 588 original plant leaf images and their corresponding binary masks, aimed at segmenting diseased versus healthy leaf regions without predefined class labels (El‐Assiouti et al. 2023; Bhatti et al. 2024). Figure 1 shows the methodology for U‐Net model design used for segmentation for leaf diseases. It starts with preprocessing tasks like data augmentation of the input dataset and then splits the data into training and test sets. To improve generalization and mitigate overfitting due to the limited dataset size, augmentation techniques were applied, expanding the dataset to 7056 images. The proposed segmentation framework is based on a modified U‐Net architecture, enhanced with attention mechanisms and identity mappings, enabling accurate localization of infected regions even under challenging conditions. The model was trained and validated using a 70:30 data split, with performance evaluated using metrics such as Dice coefficient, IoU, and accuracy. The following subsections elaborate on each component of the methodology in detail.

### 3.1 Dataset Preparation

The dataset, cited from Kaggle, contains 588 original plant leaf images and 588 corresponding binary masks, without explicit class labels or distribution across specific disease categories. As such, it is a mixed dataset, intended solely for binary segmentation of diseased vs. healthy leaf regions. To enhance model generalization and compensate for the limited sample size, we applied data augmentation techniques, which increased the total training data to 7056 images. This augmentation strategy helps prevent overfitting and improve robustness but does not introduce new class labels or alter the dataset's binary segmentation focus (Shoaib et al. 2022).

### 3.2 Data Preprocessing

**FIGURE 2:**
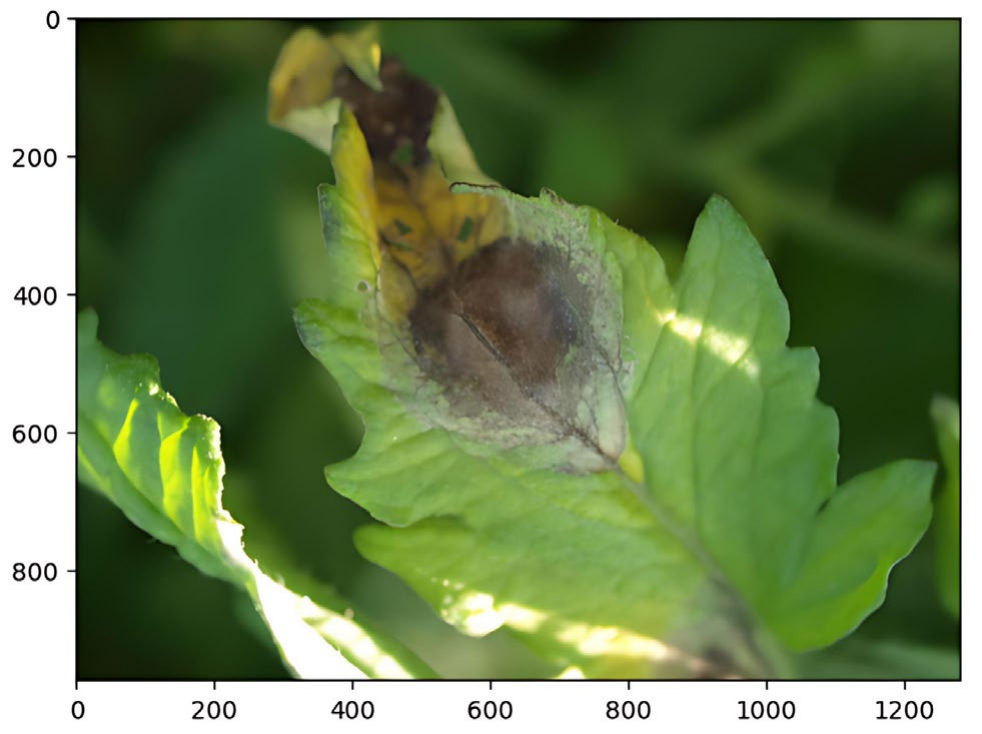
Input image.

In Figure 2, the images are a collection of high‐resolution sick plant leaf photographs along with corresponding segmentation masks. Among the numerous categories in the collection were images of leaves infected by several infested areas, including bacterial spot, early blight, and leaf mold. Each image in the dataset was scaled to a designated 256 × 256 pixel resolution, hence standardizing the input size of the U‐Net model (Dayang and Kouyim Meli 2021). This scaling directed the neural network in picture analysis and guaranteed consistency (Mzoughi and Yahiaoui 2023; Zhang et al. 2019). By scaling the pixel values between 0 and 1, the next normalization of the images helps to improve the training stability and model convergence (Elangovan and Nalini 2017). The images show a leaf with obvious infested area signs along with its matching segmented mask (Zhang and Zhang 2023). In the first picture, the leaf is shown with a clear brownish lesion surrounded by some yellowing that suggests a probable bacterial or fungal infection (Zahra et al. 2024; Lakshmi and Savarimuthu 2022).

**FIGURE 3:**
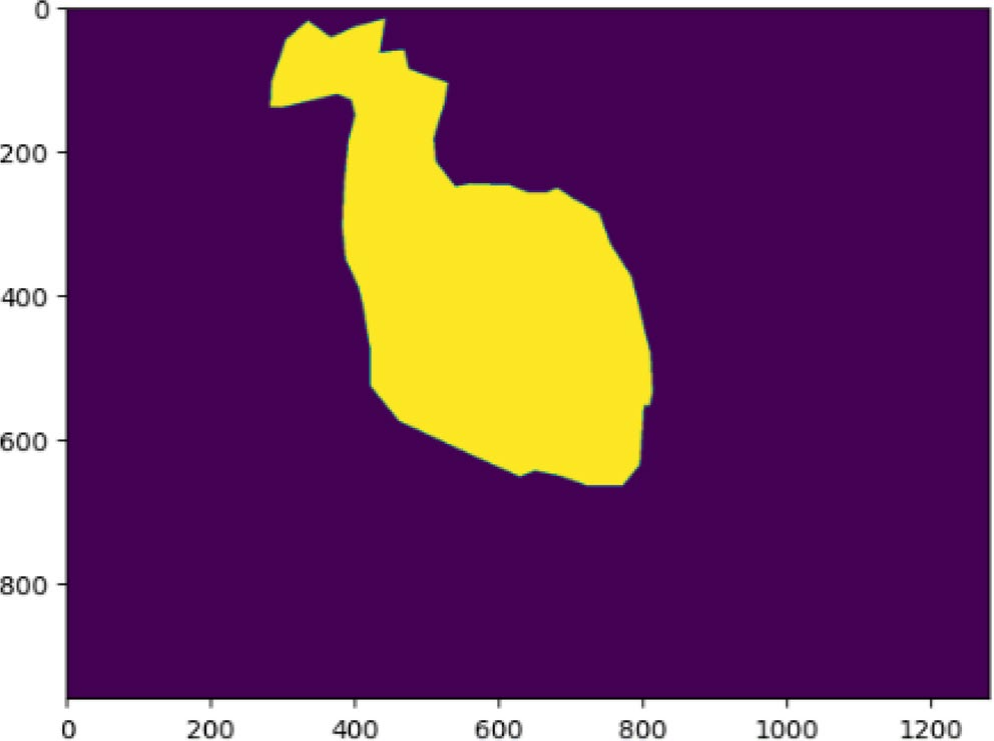
Predicted disease region mask.

The lesion is localized in the middle of the leaf, and the whole picture is crisp enough to see the variances in color and texture across the leaf surface. Figure 3 the second picture shows the segmentation mask (Zhang et al. 2019), thereby stressing the impacted leaf area. The mask is shown in yellow, precisely delineating the sick area's limits against a purple backdrop, therefore indicating the leaf's untouched areas. Analyzing the degree of the diseases depends on this segmentation, which also finds use in next phases of automated disease identification and analysis.

### 3.3 Data Augmentation

**FIGURE 4:**
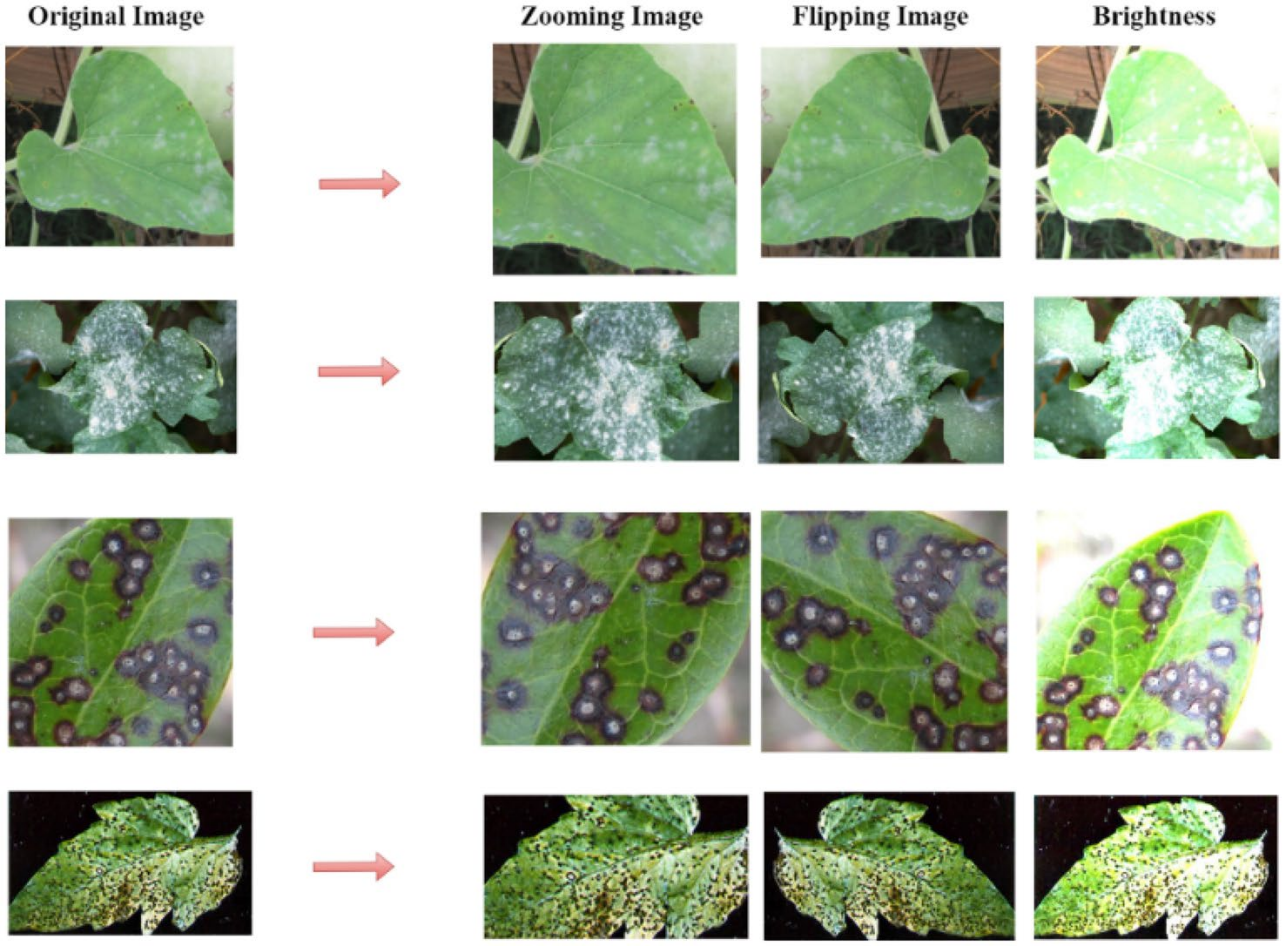
Data augmentation images samples.

Generalizing capability of the model was increased and overfitting avoided by use of data augmentation techniques. Figure 4 Part of the augmentation were horizontal and vertical flips, zooming, and brightness variations. These modifications allow the model to gain more robust features by simulating many real‐world scenarios including different light conditions and leaf orientations. By greatly expanding the dataset, augmentation produced more variation in the training samples (Rath, n.d.; Balafas et al. 2023).

### 3.4 Proposed Model

**FIGURE 5:**
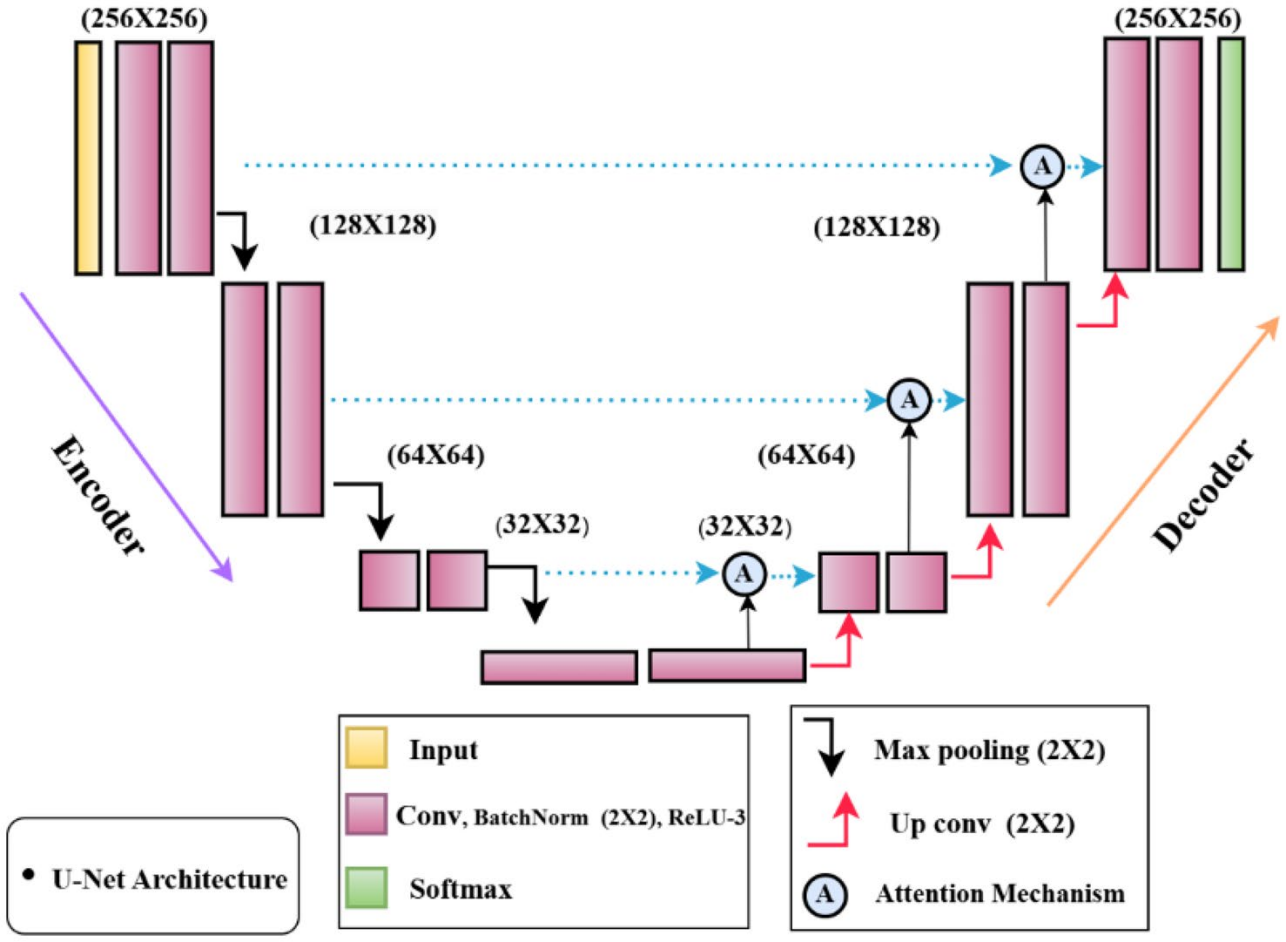
Proposed U‐Net architecture with attention mechanisms.

In Figure 5 comprising an encoder and decoder, the U‐Net design uses input images measured at 256 × 256 pixels. Moving through layers of convolution using Conv2D, BatchNorm, and ReLU activations, the image's resolution decreases; finally, max pooling compresses the spatial dimensions. The resolution falls to 128 × 128, 64 × 64, and 32 × 32 while the amount of feature maps increases at every level. Reversing this process, the decoder restores the original resolution by means of upsampling up conv. between matching levels in the encoder and decoder; skip connections shown by blue arrows help to preserve fine details. The last output is a segmentation map predicted with softmax activation whereby the model offers pixel‐wise classification of sick areas. Metrics such as Intersection over Union (IOU) and Dice score, which are emphasized in the diagram, help to evaluate the performance. Following this is a “BatchNorm2d” layer to normalize the input, hence enhancing model convergence; a “ReLU” activation function introduces non‐linearity. The architecture then comprises a “Max Pool2d” layer, therefore shrinking the spatial dimensions and so improving the model's robustness in feature recognition at different sizes. Another “Conv2d” layer with 4096 parameters is next added, then batch normalizing and ReLU activation. Repeated numerous times throughout the model, this series of convolution, batch normalization, and ReLU activation helps the model to recognize complex patterns and features pertinent to plant leaf disease diagnosis (AlArfaj et al. 2023). The U‐Net model has a noteworthy bottleneck structure. Every bottleneck consists of a ReLU activation after batch normalization and a “Conv2d” layer, therefore enabling deep representations of the input images (Chaki and Ghosh 2024; Hassan and Maji 2022). While eliminating unnecessary data, these bottlenecks are crucial in maintaining the most important aspects. The architecture of the model also consists of attention systems and identity mappings. By letting the model pass information straight across layers, the identity mappings help to preserve important information that could otherwise be lost in deeper layers (Ozcan and Polat 2025; Shwetha et al. 2024).

By weighing the relevance of different input regions, the attention mechanisms improve the model's capacity to focus on the most relevant aspects, by which this is especially important for the detection of diseases that might show subtle visual clues. Gradually scaling the feature maps back to the original input size using decoder blocks guarantees that the segmented output lines up properly with the input images. Mirroring the structure found in the encoder section of the model, each decoder block has convolutional layers, batch normalization, and ReLU activations. Designed to generate the segmentation map where every pixel in the input image is categorized as either disease or healthy, the last layers of the model are Plant leaf disease diagnosis is a job for which this U‐Net architecture, which combines convolutional layers, bottlenecks, identity mappings, and attention mechanisms, fits really well. Its architecture guarantees that local characteristics as well as global context are taken into account, therefore producing accurate and dependable segmentation of sick areas in leaf images. Based on the layer‐wise description, the general framework of the model shows a balance between depth and computing efficiency, which makes it a useful instrument for tackling the difficulties related to plant leaf disease diagnosis (*Deng* et al. 2023).

The primary novelty of this proposed hybrid U‐Net model lies in the integration of attention mechanisms and identity mappings within a modified U‐Net architecture, specifically designed for binary segmentation of diseased versus healthy plant leaf regions. Unlike traditional U‐Net models, this hybrid design incorporates attention gates that dynamically refine spatial features, enabling more accurate localization of subtle disease symptoms. Additionally, the use of identity mappings helps preserve essential features across deeper layers, thereby enhancing feature propagation and improving overall model performance. A further novel aspect of this work is the application of the model to a mixed dataset that lacks predefined class labels, focusing solely on distinguishing between healthy and infected leaf regions. This presents a more challenging and less commonly addressed segmentation task compared to typical multiclass disease classification. To address the limitation of having only 588 original images, the study employs a systematic data augmentation strategy, expanding the dataset to 7056 samples, which effectively mitigates overfitting and improves the model's generalization capabilities.

#### 3.4.1 Encoder

**FIGURE 6:**
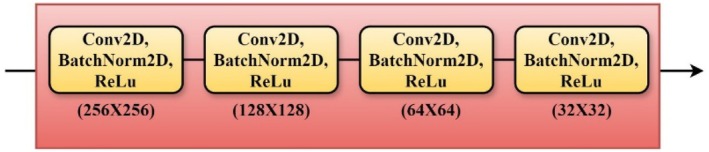
Encoder layers.

Comprising a sequence of convolutional layers and then max‐pooling layers, the encoder path, also called the contracting path, is two consecutive 2 × 2 convolutional layers with ReLU activation functions comprising each convolutional block in the encoder route; then a 2 × 2 max‐pooling layer lowers the spatial dimensions by a factor of two. After every max‐pooling operation, the number of feature channels doubles so the network may gather increasingly intricate features at each level (Deng et al. 2023; Fu et al. 2023). This down‐sampling technique enables the model to gather contextual data required for precise segmentation as depicted in Figure 6.

#### 3.4.2 Bottleneck

**FIGURE 7:**
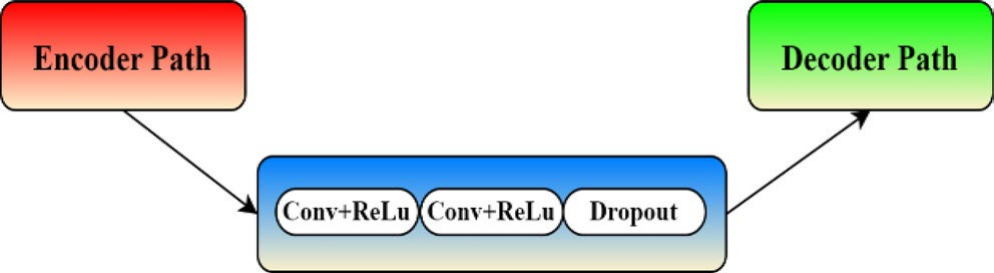
Bottleneck layer.

The bottleneck layer, located at the bottom of the U‐Net architecture, acts as a bridge between the encoder and decoder paths. It consists of two convolutional layers activated by ReLU and includes a dropout layer to prevent overfitting (Fu et al. 2023; Aslan and Özüpak 2025, 2024). This layer extracts the most abstract features from the input images, creating a condensed representation that the decoder uses to reconstruct the segmented images, as illustrated in Figure 7.

#### 3.4.3 Decoder

**FIGURE 8:**
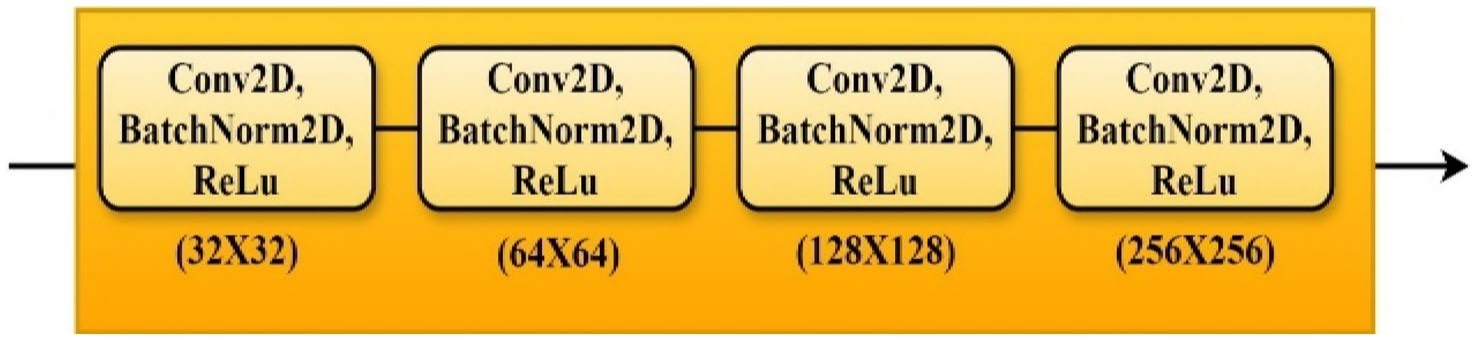
Decoder layers.

Although it uses up‐sampling rather than down‐sampling, the decoder or expanding path mirrors the encoder path. As shown in Figure 8, every convolutional block in the decoder comprises an up‐sampling layer that doubles the spatial dimensions of the feature maps, then is concatenated with the matching feature maps from the encoder path (Deng et al. 2023; Fu et al. 2023).

This skip connection approach guarantees that up‐sampled feature maps mix with fine‐grained encoder details, hence preserving them. Two 2 × 2 convolutional layers with ReLU activations are used to enhance the segmentation map following concatenation. The last output layer transfers the feature maps to the intended number of output channels using a 1 × 1 convolution, therefore matching the number of classes in the segmentation task—that is, both diseased and healthy areas (Aslan and Özüpak 2025).

#### 3.4.4 Attention Mechanisms

In the proposed hybrid U‐Net architecture, attention mechanisms are integrated to enhance the network's ability to focus on relevant regions of the input image while suppressing less informative features. Specifically, attention gates are employed in the skip connections to refine the features passed from the encoder to the decoder. These gates learn to highlight salient spatial regions associated with disease symptoms, allowing the model to make more precise segmentation decisions. By dynamically weighting feature maps based on contextual relevance, the attention mechanism improves localization performance, particularly in challenging cases where disease patterns are small or dispersed. This modification enables the model to better distinguish between healthy and diseased leaf regions, contributing to improved segmentation accuracy.

### 3.5 Training Procedure

Preprocessing the dataset, which involves downsizing input photos and normalizing pixel values, is the first step in the training process for the suggested U‐Net model. The robustness of the model is then improved by using data augmentation methods to broaden the variety of the training data. The Adam optimizer and a learning rate scheduler that modifies learning rates dynamically during training are used to fine‐tune the U‐Net model once it has been initialized with pre‐trained weights. The unminimized loss function maximizes segment accuracy to minimize the overlap error between ground truth masks and predicted performance parameters, including accuracy, IoU, and Dice coefficient, are monitored throughout many epochs when the dataset is divided into training and validation sets (Aslan and Özüpak 2024). A short explanation of the hyperparameter values utilized during model training is provided in Table 2.

**TABLE 2:** Hyperparameter settings for training the proposed U‐net‐based segmentation model.

Hyperparameter	Value
Image size	256 × 256
Optimizer	Adam
Initial learning rate	0.001
Batch size	32
Epochs	100
Loss function	Dice loss + binary cross‐entropy
Dropout rate	0.5
Regularization	Early stopping, data augmentation
Augmentation techniques	Flipping, zoom, brightness

#### 3.5.1 Loss Function

Training the U‐Net model proved possible by combining the Dice coefficient loss with the binary cross‐entropy (BCE) loss. Pixel‐wise classification accuracy is estimated by way of a comparison between the ground truth mask and the projected segmentation mask. On the other hand, the Dice coefficient loss indicates the overlap between the actual and expected masks, thereby highlighting the appropriate segmentation of smaller sick areas—necessary for the early‐stage disease diagnosis (Shwetha et al. 2024; Deng et al. 2023). Together, the loss function assures not only exact pixel categorization but also produces a coherent segmentation map quite similar to the real sick areas.

#### 3.5.2 Optimizer

Adam optimizer's computational efficiency and flexible learning rate led us to choose it for training. Beginning at 0.001, the learning rate declined under an exponential decay schedule over training. This method helped the model converge to an optimal solution by stepping larger in the first phases of training and finer steps as it approaches the least loss.

#### 3.5.3 Training and Validation Split

Training and validation sets made up a 70:30 split from the dataset. The proposed U‐Net model was trained on the training set for 100 epochs; performance of the model was tracked, and halting overfitting was achieved using the validation set. The training was done over more than 100 epochs, using early stopping to halt should the validation loss not rise for 10 consecutive epochs. This ensured that the model maintained itself from overfitting to the training set and correctly generalized to raw input.

### 3.6 Evaluation Metrics

The proposed U‐Net model was evaluated using pixel accuracy, dice coefficient, and IoU among several criteria. These tests provided a full assessment of the model's ability to precisely separate diseased regions of the leaves from their healthy counterparts. IoU computes the overlap between the anticipated and ground truth masks, while the Dice coefficient measures their similarity. Pixel accuracy gauges the entire accurately classified pixel count of the image. These values calculated for the validation and training sets track the performance of the model all through the training process. Set the epoch to 100, but getting a better IoU score and lowest Dice loss values on epoch 40.

The mathematical formula for the IoU score is:

(1) $$\mathrm{IoU}=\frac{|P⋂G|}{|P⋃G|}$$

where *P* is the set of predicted pixels. *G* is the set of ground truth pixels. ∣*P*∩*G*∣ represents the number of pixels where the predicted and ground truth segmentations overlap. ∣*P*∪*G*∣ represents the total number of pixels in both the predicted and ground truth segmentations.

This can also be written as:

(2) $$\mathrm{IoU}=\frac{\sum_{i=1}^{N}\text{pigi}}{\sum_{i=1}^{N}\left(\mathrm{pi}+\mathrm{gi}-\text{pigi}\right)}$$

where *pi* is the predicted binary value for pixel *i*. *gi* is the ground truth binary value for pixel *i*. *N* is the total number of pixels.

The mathematical formula for the Dice coefficient is:

(3) $$\text{Dice Coefficient}=\frac{2\sum_{i=1}^{N}\text{pigi}\;}{\sum_{i=1}^{N}\mathrm{pi}+\sum_{i=1}^{N}\mathrm{gi}}$$

where *pi* represents the predicted binary pixel values (0 or 1) for pixel *i*. *gi* represents the ground truth binary pixel values (0 or 1) for pixel *i*. *N* is the total number of pixels.

The Dice Loss is calculated as:

(4) Dice Loss=1−Dice Coefficient

(5) $$\text{Dice Loss}=1-\frac{2\sum_{i=1}^{N}\text{pigi}\;}{\sum_{i=1}^{N}\mathrm{pi}+\sum_{i=1}^{N}\mathrm{gi}}$$

## 4 Result and Discussion

The proposed U‐Net model was implemented using Python and Keras, which offer a robust and flexible framework for developing, training, and evaluating DL models. Training was conducted on a machine equipped with an NVIDIA GPU, significantly accelerating computation and enabling efficient experimentation and model refinement. Post‐processing techniques were applied to improve segmentation mask quality, including morphological operations such as dilation and erosion to remove noise and small artifacts that could be mistaken for infected regions. Connected component analysis was used to isolate and highlight only significant diseased areas. For evaluation, the model was tested on an independent test set containing a diverse range of leaf images to assess performance under various conditions. Results were compared against expert‐annotated ground truths provided by agricultural professionals, ensuring practical relevance in real‐world applications (Fu et al. 2023).

### 4.1 IoU and Dice Loss Results

**FIGURE 9:**
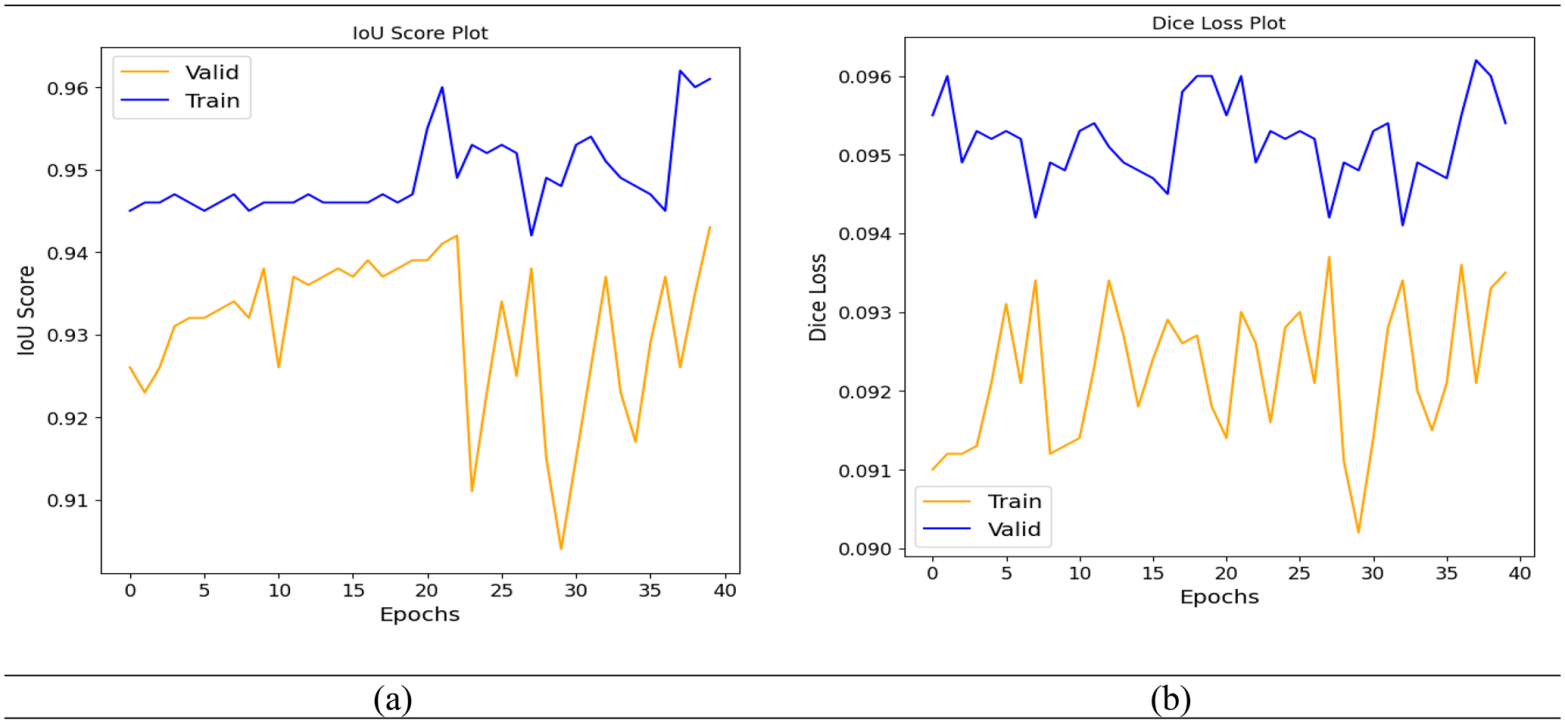
Model performance measures during training and validation (a) IoU score and (b) Dice loss.

The Intersection over Union IoU Score graph shown in Figure 9a the evolution of the IoU metric for both training and validation datasets over 40 epochs. IoU measures the overlap between the predicted and actual segmentation regions, making it a crucial metric for image segmentation tasks. The training IoU remains consistently high, ranging between 0.94 and 0.96. This suggests that the model is accurately learning to segment the disease regions in the training images. The validation IoUstarts slightly lower than the training IoU and gradually increases until around epoch 20. After that point, the validation IoU becomes more erratic, with visible fluctuations between 0.91 and 0.94, indicating some instability or variance in generalization to unseen data. Despite the fluctuations, the validation IoU still stays relatively close to the training IoU, and both maintain high values overall. This indicates that while the model performs well, further improvements such as regularization, better data augmentation, or fine‐tuning might help stabilize validation performance and reduce variance across epochs.

The Dice loss graph shown in Figure 9b presents the training and validation loss performance of the proposed hybrid U‐Net model across 40 epochs. The Dice loss, a metric that quantifies the overlap between predicted segmentation masks and ground truth, is a key indicator of model accuracy in medical and plant image segmentation tasks, with lower values indicating better performance. In this graph, Figure 9b the training Dice lossremains relatively low and stable, fluctuating between 0.090 and 0.094. This indicates that the model is effectively learning to segment the disease‐affected regions in the training dataset. On the other hand, the validation Dice lossremains consistently higher than the training loss, hovering around 0.094–0.096. Despite these fluctuations, both curves maintain relatively low Dice loss values overall, indicating that the model exhibits good segmentation capability.

Table 3 presented the performance of the proposed hybrid U‐Net model this is evaluated across four key epochs (10, 20, 30, and 40) using two primary segmentation metrics IoU and Dice loss along with their respective SDs to assess stability and reliability. At epoch 10, the training IoU reaches 0.946, and the validation IoU is slightly lower at 0.938, indicating the model is learning to segment disease regions with reasonable generalization. The corresponding Dice loss values are 0.0948 (training) and 0.0913 (validation), with standard deviations of 0.0035 and 0.0021, respectively. This low variability suggests that the model's performance is consistent across batches. By epoch 20, the IoU values slightly improve to 0.947 (training) and 0.939 (validation), and the Dice Loss shows a marginal increase in training (0.096) and validation (0.0918), indicating a steady learning phase. The standard deviations (IoU: 0.0029, Dice Loss: 0.0023) confirm stable training. However, at epoch 30, despite a rise in training IoU to 0.948, validation IoU drops to 0.904. Still, Dice loss remains low (0.0948 training, 0.0902 validation), and the standard deviation in IoU increases to 0.0041, reflecting instability.

**TABLE 3:** Training and validation IoU score and dice loss values.

Epoch	Training IoU	Validation IoU	Training dice loss	Validation dice loss	IoU SD	Dice loss SD
10	0.946	0.938	0.0948	0.0913	0.0035	0.0021
20	0.947	0.939	0.096	0.0918	0.0029	0.0023
30	0.948	0.904	0.0948	0.0902	0.0041	0.002
40	0.961	0.943	0.0954	0.0935	0.0027	0.0022

By epoch 40, the training IoU improves significantly to 0.961 and validation IoU recovers to 0.943. Although the Dice loss increases slightly (training: 0.0954, validation: 0.0935), the standard deviations (IoU: 0.0027, Dice: 0.0022) suggest stable convergence. Overall, the model demonstrates strong segmentation performance with minor fluctuations that warrant attention to regularization for improved generalization.

### 4.2 Accuracy and Loss Results

**FIGURE 10:**
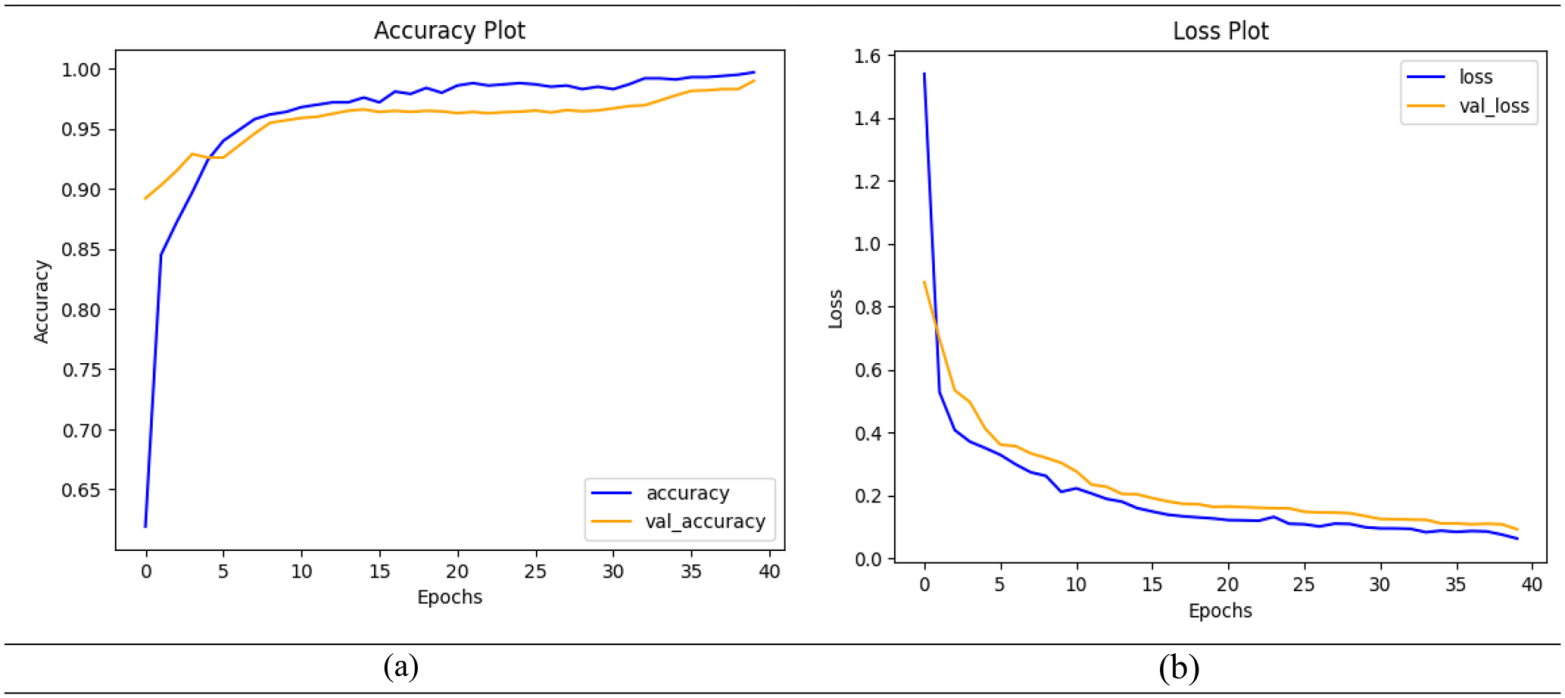
Model performance measures during training and validation (a) accuracy and (b) loss.

A DL model's training and validation accuracy across 40 epochs is shown in the accuracy graph in Figure 10a above. The graph shows that training and validation accuracy improved significantly during the early epochs (1–5), suggesting that the model rapidly picks up broad patterns from the data. The training accuracy gradually increases after epoch 10 and reaches 100%, indicating that the model fits the training data well. The validation accuracy increases first before stabilizing at 96%–97%, starting at epoch 15. The model seems to be generalizing effectively and not overfitting if the validation accuracy stays around the training accuracy and does not drastically drop. A well‐trained model should exhibit consistent performance across visible (training) and unseen (validation) data, as shown by the narrow gap between the curves.

The loss graph in Figure 10b illustrates how the training and validation loss change over 40 epochs during model training. At the beginning (epoch 0–5), both training and validation loss decrease sharply, demonstrating that the model is quickly learning and minimizing errors on both seen and unseen data. This steep decline shows effective early learning. As training continues, the loss values for both sets gradually reduce and converge closer to zero, which reflects improved prediction accuracy. After around epoch 15, the loss values become more stable with minimal fluctuations. The training loss keeps decreasing and approaches nearly zero by epoch 40, showing the model well fits the training data. The validation loss also continues to decline but remains slightly higher than the training loss throughout, suggesting mild overfitting but still within an acceptable range. Importantly, there is no significant gap or sudden rise in validation loss, which means the model generalizes well and is not severely overfitting. Overall, the graph confirms that the model is learning effectively, maintaining low error rates, and balancing performance across both training and validation datasets.

**FIGURE 11:**
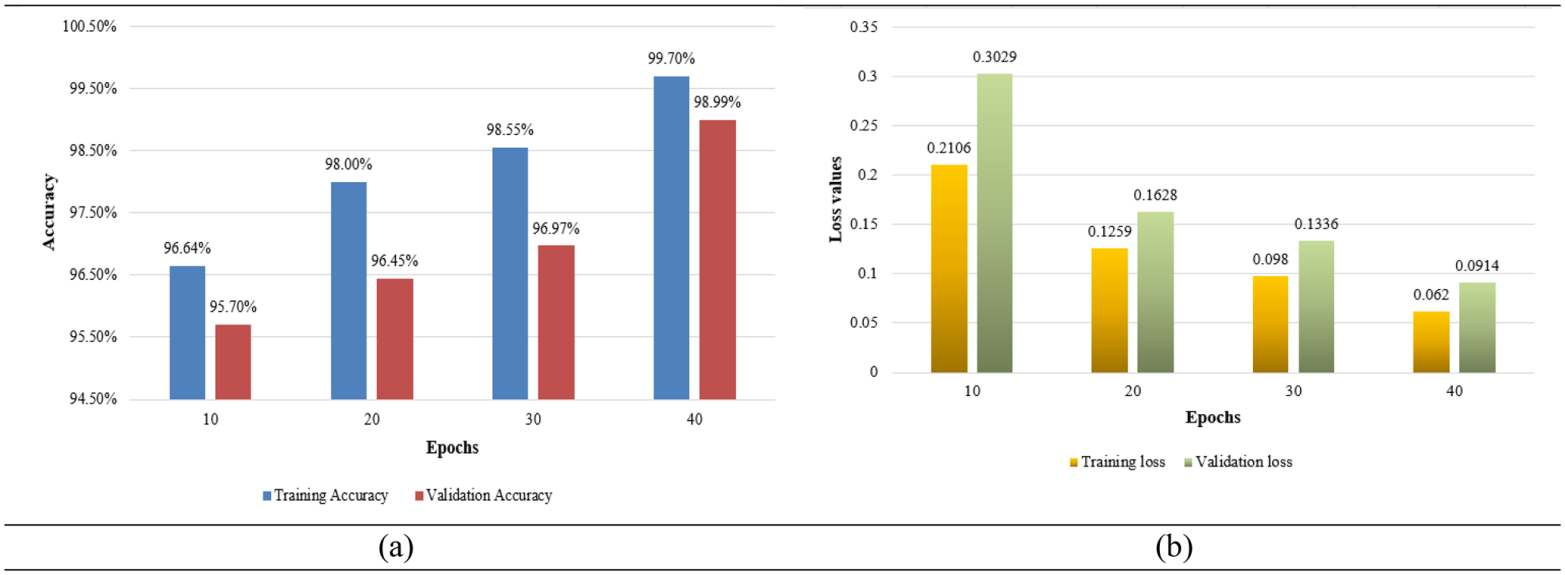
Model performance measures during training and validation (a) accuracy and (b) validation.

In Figure 11a, the bar graph demonstrates the training accuracy of a model at various epochs; it progresses from epoch to epoch. In fact, the training accuracy is 96.64% at 10 epochs and increases to 98.00% at 20 epochs, and it again increases to 98.55% at 30 epochs. The highest accuracy of 99.70% is achieved after 40 epochs, indicating that the model continues to learn and fit the training data better over time. The accuracy gains are significant initially (from 10 to 20 epochs) but gradually diminish, reflecting the typical behavior of a model nearing its performance saturation, showing an increase with an increase in epochs. At 10 epochs, the validation accuracy is 95.70%, which increases to 96.45% after 20 epochs and then to 96.97% after 30 epochs. The maximum validation accuracy of 98.99% is obtained after 40 epochs, meaning that the model generalizes better with more training. The improvement is more significant in earlier epochs (10–20) and slows down in later stages, which is typical as the model approaches optimal performance on the validation set.

In Figure 11b, the bar graph shows the training loss of a model at different epochs. The loss decreases consistently with an increase in the number of epochs. For 10 epochs, the training loss is 0.2106, which is significantly reduced to 0.1259 after 20 epochs and further drops to 0.098 at 30 epochs. The lowest training loss is 0.062, achieved at 40 epochs; this shows that the model continues to become more and better at minimizing the errors on the training dataset. The steady decline in training loss depicts effective learning where the reduction keeps diminishing as the number of epochs increases, a common trend in convergence. The bar graph represents the model's validation loss at different epochs; when the epoch value increases, a constant decline trend is observed throughout the training of the model. A validation loss at 10 epochs is 0.3029, which reduced significantly to 0.1628 after training for 20 epochs, and a further reduction occurred at 30 epochs to become 0.1336, and the validation loss is further reduced to 0.0914 with 40 epochs. The trend is decreasing, which shows better generalization and fewer errors, with more significant improvements in the earlier epochs and smaller reductions as the model converges.

### 4.3 Results on Epoch 40

**FIGURE 12:**
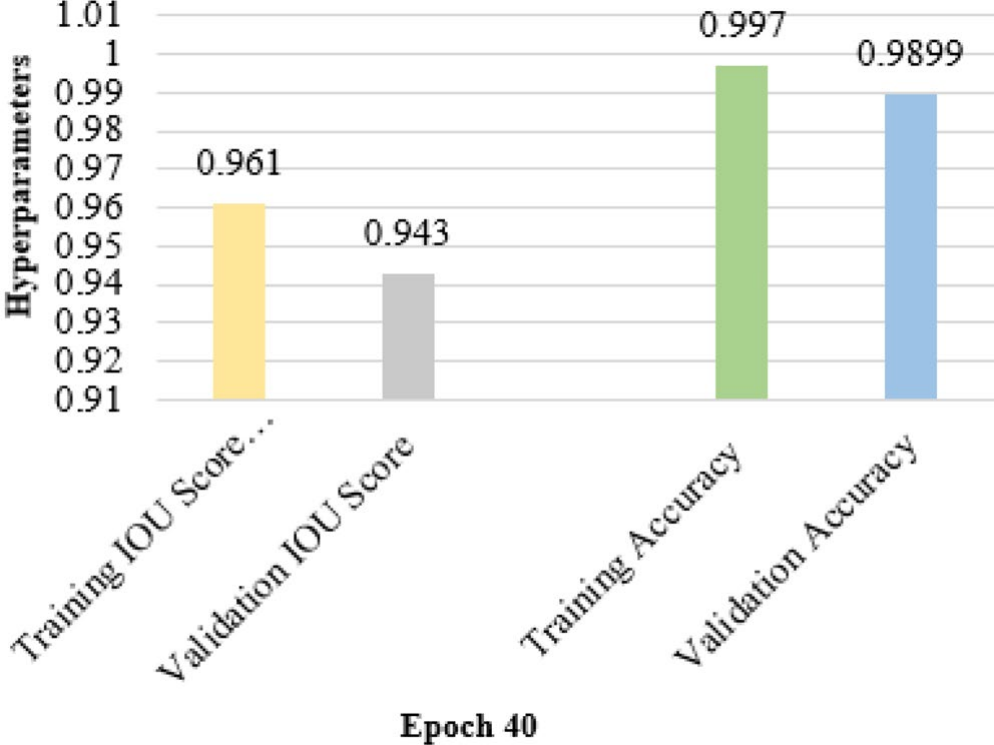
IOU and accuracy values on epoch 40.

In Figure 12, the bar chart illustrates the performance metrics for a model after 40 training epochs, focusing on IOU scores and accuracy values for both training and validation phases. The training IOU score reaches 0.961, while the validation IOU score is slightly higher at 0.943, indicating good model generalization for segmentation tasks.

The rather high training accuracy of 0.9970 indicates that the model can effectively learn from the training data. With a validation accuracy of 0.9899, the model shows that performance marginally decreases when compared to the training period even if it performs well on unseen data.

This difference can indicate some degree of overfitting or the need for further hyperparameter or regularizing approach tweaking. Generally, the measurements show that the model has performed pretty well, especially in training; on validation data, it has a solid, though slightly lower, performance.

**FIGURE 13:**
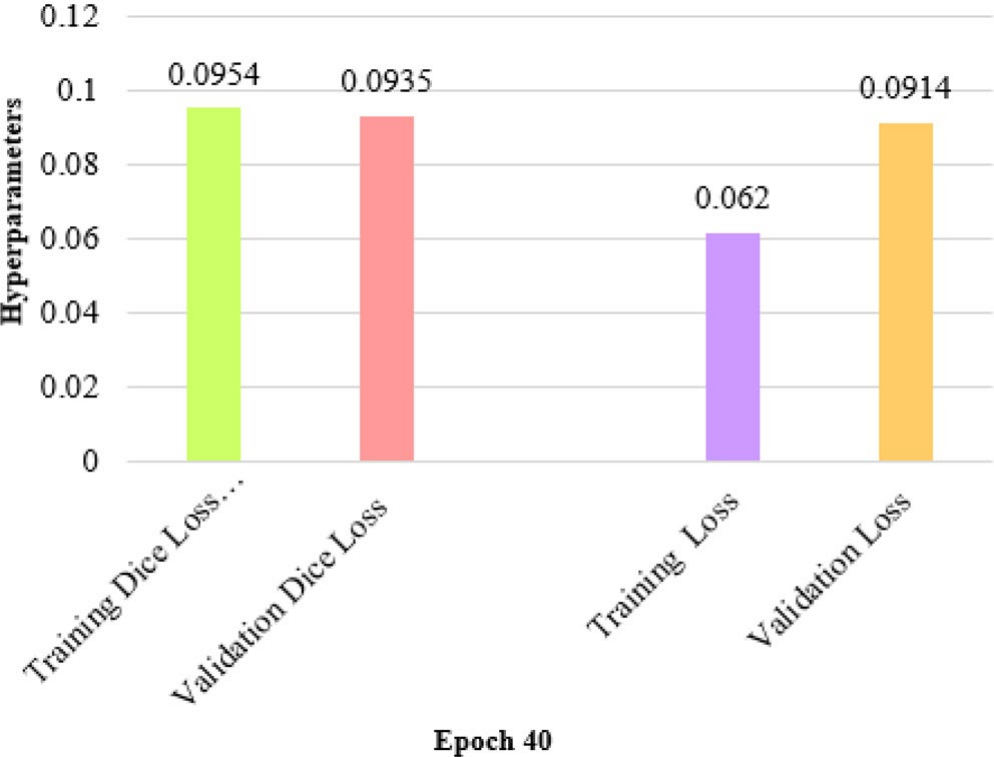
Dice loss and loss values on epoch 40.

In Figure 13, a bar chart compares the loss metrics of the proposed model after 40 epochs, highlighting both training and validation performance in terms of Dice loss and overall loss. The training Dice loss stands slightly higher at 0.0954, while the validation Dice loss is 0.0935, indicating a minor gap between the two phases. This small gap suggests that the model maintains good generalization in segmentation performance across training and validation datasets. The overall training loss is relatively low at 0.062, reflecting the model's effectiveness in reducing training errors. However, the validation loss is significantly higher at 0.0914, indicating a more considerable disparity between the two loss values. This disparity suggests the presence of potential overfitting, where the model performs significantly better on the training data compared to unseen validation samples. Such results may require adjusting regularization techniques or tuning hyperparameters to reduce overfitting and improve the model's generalization capability. Overall, the loss metrics reflect good model performance with some room for improvement on validation data.

### 4.4 Visualization of Predicted Result

**FIGURE 14:**
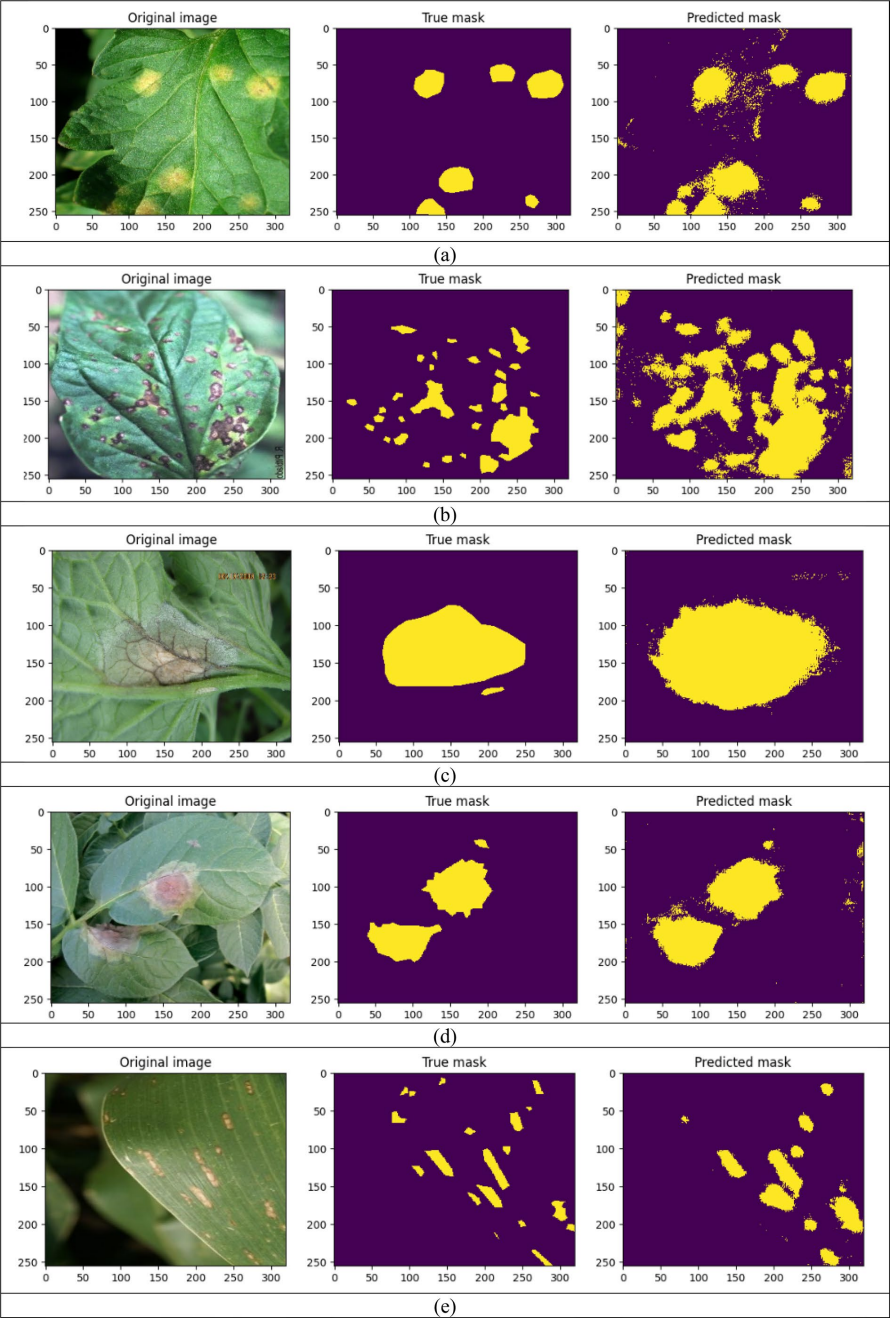
Visualization of predicted result.

This section displays the predicted results along with the mask obtained during testing of the model. Figure 14 set presents side‐by‐side comparisons of original leaf images, their corresponding ground truth masks, and the predicted masks generated by the proposed U‐Net‐based segmentation model during testing. The true masks effectively highlight the diseased regions in yellow against a purple background. The predicted masks closely resemble the ground truths in several cases, capturing the major infected regions. However, some images show over‐segmentation or scattered predictions around the edges, particularly in complex patterns with multiple small lesions. Despite these challenges, the model demonstrates a strong ability to identify the diseased areas, showcasing its potential for practical plant disease segmentation applications.

### 4.5 State‐of‐Art Comparison

The comparative analysis of accuracy and IoU performance of recent image segmentation models for plant disease detection for Leaf diseases segmentation dataset is highlighted in Table 4. In 2023, the class activation maps (CAM) approach (Uysal et al. 2023) achieved a moderate accuracy of 0.9350 but a relatively low IoU of 0.4518, indicating limited precision in pixel‐wise segmentation. In contrast, the VMC‐Unet model (Liu et al. 2023), introduced in 2024, demonstrated substantial improvements with an accuracy of 0.9782 and a much higher IoU of 0.8675, reflecting better localization of diseased areas. Similarly, the Advanced CNN Segmentation method (Shi et al. 2024) achieved 0.9580 accuracy and an IoU of 0.8605, showing reliable segmentation capabilities. The CS‐Net, a Conv‐Simpleformer‐based model (Şener and Ergen 2024), delivered a balanced performance with 0.9500 accuracy and a robust IoU of 0.8970. Notably, the Proposed U‐Net Model outperformed all other methods, achieving an outstanding 0.9899 accuracy and 0.9430 IoU, confirming its effectiveness in segmenting plant disease regions with high precision and generalization.

**TABLE 4:** State of the art of existing image segmentation techniques and models.

Years	Ref	Image segmentation technique/model	Accuracy	IOU score
2023	Uysal et al. (2023)	Class Activation Maps (CAM)	0.9350	0.4518
2023	Liu et al. (2023)	CS‐Net (Conv‐Simpleformer Network)	0.9500	0.8970
2024	Shi et al. (2024)	VMC‐Unet	0.9782	0.8675
2024	Şener and Ergen (2024)	Advanced CNN Segmentation	0.9580	0.8605
Proposed U‐Net Model			0.9899	0.9430

## 5 Conclusion

This paper presents the pixel‐wise segmentation performance of the U‐Net architecture for the purpose of leaf disease detection. The method efficiently identifies diseased regions using a well‐annotated leaf image dataset, highlighting its potential for precision agriculture. Through the application of data augmentation, batch normalization, and dropout, our approach improves segmentation accuracy and generalization. The U‐Net model accurately recognizes and isolates affected leaf areas, outperforming conventional image processing techniques even under challenging test conditions. Early diagnosis of plant diseases is essential for timely intervention, better crop management, and yield preservation, and our method offers a scalable and automated solution for achieving these objectives.

In addition to its accuracy, the proposed model is computationally efficient and suitable for real‐time deployment on edge devices such as mobile GPUs or AI‐integrated agricultural drones. The model requires moderate training resources but has low inference‐time costs, making it viable for widespread use in practical farming scenarios. This balance between cost and performance strengthens the real‐world applicability of the method.

### 5.1 Limitations of the Proposed Work

While the proposed U‐Net‐based segmentation model demonstrates excellent performance on the given dataset, achieving high accuracy and IoU scores, several limitations must be acknowledged. First, the model has been trained and evaluated on a single mixed dataset containing a specific set of crop disease images. As such, its generalization to unseen crops or rare disease patterns remains uncertain. Additionally, since the dataset images are captured under relatively consistent lighting and background conditions, the model may struggle to maintain performance when applied to real‐world field scenarios with varying illumination, shadowing, occlusion, or cluttered backgrounds. These limitations suggest that the model can be trained on more diverse and multi‐labeled datasets and real‐time augmentation in the future to enhance generalization and deployment readiness.

## Author Contributions

**Gurpreet Singh:** conceptualization (equal), methodology (equal), software (equal), writing – original draft (equal). **Asma A. Al‐Huqail:** conceptualization (equal), methodology (equal), project administration (equal), resources (equal), supervision (equal), writing – review and editing (equal). **Ahmad Almogren:** conceptualization (equal), project administration (equal), software (equal), supervision (equal), writing – review and editing (equal). **Sukhdeep Kaur:** data curation (equal), formal analysis (equal), visualization (equal). **Kapil Joshi:** investigation (equal), validation (equal), visualization (equal). **Ajay Singh:** formal analysis (equal), investigation (equal), visualization (equal). **Salil Bharany:** formal analysis (equal), investigation (equal), validation (equal). **Seada Hussen:** conceptualization (equal), methodology (equal), writing – review and editing (equal). **Ateeq Ur Rehman:** conceptualization (equal), methodology (equal), project administration (equal), writing – review and editing (equal).

## Conflicts of Interest

The authors declare no conflicts of interest.

## Data Availability

The dataset used in this study, the “Leaf Disease Segmentation Dataset,” is publicly available on Kaggle and can be accessed at https://www.kaggle.com/datasets/fakhrealam9537/leaf‐disease‐segmentation‐dataset.
